# The Type of Conservative Management Could Be Related to the Strength of the Inspiratory Muscles of Adolescents with Idiopathic Scoliosis—A Case Series

**DOI:** 10.3390/children8111002

**Published:** 2021-11-03

**Authors:** Felipe León-Morillas, Silvana Loana de Oliveira-Sousa, Juan Alfonso Andrade-Ortega, Alfonso Javier Ibáñez-Vera, Rafael Lomas-Vega, Noelia Zagalaz-Anula

**Affiliations:** 1 Department of Physiotherapy, Catholic University of Murcia UCAM, Avenida de los Jerónimos, 30107 Murcia, Spain; fleon@ucam.edu; 2 Department of Physiotherapy, University of Murcia, 30100 Murcia, Spain; soliveira@um.es; 3 Department of Physical Medicine and Rehabilitation, Complejo Hospitalario de Jaen, Avenue of Madrid, 23001 Jaén, Spain; juanalfonsoandrade@gmail.com; 4 Department of Health Sciences, University of Jaen, Campus de las Lagunillas, 23071 Jaén, Spain; rlomas@ujaen.es (R.L.-V.); nzagalaz@ujaen.es (N.Z.-A.)

**Keywords:** idiopathic scoliosis, respiratory muscle strength, lung function, global posture reeducation

## Abstract

Adolescent idiopathic scoliosis (AIS) is a lateral curvature of the spine with a Cobb angle of at least 10° with an unknown etiology. It is recognized that AIS may affect respiratory function. This study aims to describe and compare respiratory function in a case series of patients with scoliosis who underwent different types of therapeutic management: no intervention, orthotic brace, and global postural reeducation (GPR). Fifteen AIS patients were included in this study (seven no intervention, four orthotic brace and four GPR). Lung function and inspiratory muscle strength were measured and analyzed, as well as sociodemographic, clinical, and anthropometric variables. Significant correlations were observed between height (cm) and maximum inspiratory pressure (MIP) reference (cmH_2_O) and forced vital capacity (FVC) (liters) (*r = 0*.650 and *r* = 0.673, respectively; *p* < 0.01); weight (Kg) and MIP reference (cmH_2_O) (*r* = 0.727; *p* < 0.01); and Main curve degrees (Cobb angle) and FVC% (*r* = −0.648; *p* < 0.01). The AIS cases that underwent GPR treatment presented a greater MIP (% predictive) compared to the no intervention and brace cases (201.1% versus 126.1% and 78.4%, respectively; *p* < 0.05). The results of this case series show a possible relation whereby patients undergoing treatment with the GPR method have greater inspiratory muscle strength compared to the no intervention and brace cases. Studies with larger samples and prospective designs must be performed to corroborate these results.

## 1. Introduction

The process of ossification and volumetric growth of the spine is long-lasting, beginning in the third month of intrauterine life and lasting until the second decade of life. More than 130 growth plates working in perfect synchronization are involved in spinal growth. Idiopathic scoliosis (IS) is an evolutive growth plate disorder that produces negative effects on the growing spine [1]. Puberty is a turning point in children with IS as the pubertal growth spurt increases the risk of deformity progression [1]. Adolescent idiopathic scoliosis (AIS) is the most common form of scoliosis in children between 10 and 18 years of age (80–85% of recorded cases). AIS is characterized by an unknown etiology and a lateral deviation by a Cobb angle of at least 10° [2,3].

It is recognized that AIS may affect respiratory function. AIS reduces diaphragmatic movement and generates an uneven distribution of inhaled air, which is why it can affect lung function in these patients [3,4]. Several authors have shown a direct correlation between lung disability and the magnitude of the degree of deviation of the spine [5,6]. At Cobb angles greater than 60°, patients present severe functional restrictions in breathing [7]. Those with curvatures between 20° and 45° may find their maximum ventilatory capacity to be limited, manifesting in difficulties carrying out intense physical exercise [8]. Redding et al. also mentioned the relationship between forced vital capacity and Cobb angle in patients with AIS [9]. For this reason, correction and respiratory treatment in these patients are essential.

The management of scoliosis mainly depends on age (remaining growth), pattern of curve, severity of the deformity, risk of progression, and presence of comorbidities. Specifically, the remaining growth is especially important in the worsening of the curvature of the spine, since the risk of progression of the curve is greater in younger children. Furthermore, the pubertal growth spurt increases the risk of deformity progression with a significant number of cases substantially worsening [10]. Almost 10% of patients require some type of treatment and 0.1% end up undergoing surgery [8]. The scientific literature is in agreement that with appropriate indications—still-growing and well-documented progressive curves between 20° and 45°—a well-designed and adapted brace can provide a correction of 50%, which can stop the curve’s progression in most cases [8]. However, the effects of bracing on thorax mechanics, chest mobility, and pulmonary functions are considered inevitable [11]. Other conservative methods, based on exercise approaches, such as Schroth exercises [12], or Global Postural Reeducation (GPR) [13], may be beneficial and appear to cause fewer adverse effects because they allow the patient to move without restriction. Specifically, the GPR uses active muscle stretching postures, motor control and sensory integration exercises. The reduction in the scoliotic curve after GPR treatment has been measured during GPR active and assisted self-correction [13].

Some studies based on exercise approaches have shown reductions in Cobb angle and improvement in chest expansibility, vital capacity and respiratory muscle strength [12,14,15,16,17]. However, although these are promising therapies for the management of scoliosis, the available evidence is insufficient [12], especially with respect to the effects on respiratory function. Therefore, the objective of this study is to describe and compare lung function and inspiratory muscle strength in patients with AIS, under three different approaches to therapeutic management: no intervention, orthotic brace and GPR.

## 2. Materials and Methods

### 2.1. Participants

The design of this study was a retrospective case series. Patients with AIS aged between 10 and 18 years were treated between 10 September 2018 and 13 May 2019 at the Tertiary Hospital of Jaén (Andalusia, Spain) or at specialized private clinics or health centers (any with both rehabilitation doctors and physiotherapist in their staff) in the province we selected. Inclusion criteria were a Cobb angle greater than 10° (which was assessed attending King Classification of AIS) and undergoing conservative treatment or no intervention. Patients with neuromuscular problems and surgical treatment were excluded. The study was designed and conducted in accordance with the World Medical Association Code of Ethics for studies with human participants (Declaration of Helsinki). The research protocol was approved by the Hospital de Jaén Ethics Committee (Code 0594-N-19, date of approval 30 May 2019). All of the participants were informed and voluntarily decided to participate in the study together with the agreement and consent of their legal guardians.

### 2.2. Therapeutic Management

**Figure 1:**
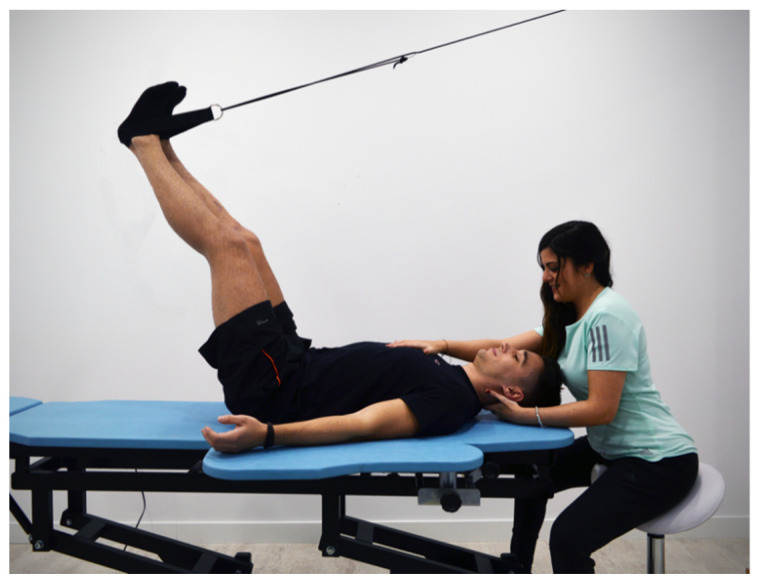
Example of GPR therapeutic management, with patient placed in lying position with lower limbs hanged to increase the tension in the triceps surae, hamstring muscles and gluteus maximus while asked to perform paradoxical breathing and spine corrections. The posture will evolve to increase in hip flexion.

**Figure 2:**
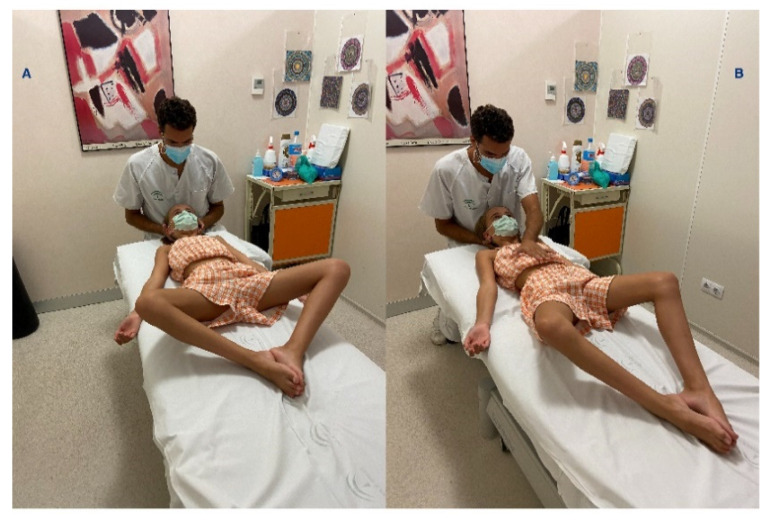
Example of GPR therapeutic management, with patient placed in lying position with lower limbs in closed chain position to increase the tension of the psoas muscles while asked to perform paradoxical breathing and spine corrections (**A**). The posture will evolve to reduction in hip flexion (**B**).

On the one hand, patients with moderate curvature (20–45 degrees) were treated 8.25 ± 2.86 months (mean ± standard deviation) with a Cheneau Brace, a type of orthosis effective in the treatment of scoliosis [18]. On the other hand, patients who presented with slight curvature (10–19 degrees) performed GPR exercises twice a month for 6.25 ± 2.95 months. This management consisted of maintaining the anteroposterior curves (the physiological kyphosis and lordosis) and correcting the lateral curves of the spine through a self-stretching and a “paradoxical” breathing (active descent of the diaphragm and abdominal and oblique contraction at the end of expiration) that increase the stretching at the affected side. These exercises could be performed with legs hung to increase the tension in the triceps surae, hamstring muscles and gluteus maximus (Figure 1) or with legs in closed chain to increase the tension of the psoas muscles (Figure 2), depending on a previous evaluation. The postures always evolve towards a tension increment by reducing hip angle or increasing hip angle, respectively, avoiding any compensation al lumbar spine. The GPR exercise reduces the asymmetry through muscle eccentric stretching guided by breathing and sensory inputs directed by the therapist [13].

### 2.3. Measurements

The diagnosis of AIS was made by a physician specializing in Physical Medicine and Rehabilitation. Cobb angle was measured to be greater than or equal to 10° through anteroposterior X-ray. There are five types of scoliotic curve, according to the King Classification [19] of AIS: “S-shaped curve” in which both the thoracic curve and the lumbar curve cross midline and lumbar curve larger than thoracic curve on standing roentgenogram (Type I); “S-shaped curve” in which the thoracic curve and the lumbar curve cross the midline. Thoracic curve ≥ lumbar curve (Type II); the thoracic curve in which the lumbar curve does not cross the midline (Type III); long thoracic curve in which the fifth lumbar vertebra is centered over the sacrum but the fourth lumbar vertebra tilts into the thoracic curve (Type IV); the double thoracic curve with the first thoracic vertebra tilted into the convexity of the upper curve. The upper curve structural on side-bending (Type V). Sociodemographic, clinical, and anthropometric variables were collected and written down by the same researcher in all of the patients at the same time point, regardless of the time of evolution of the treatments.

**Figure 3:**
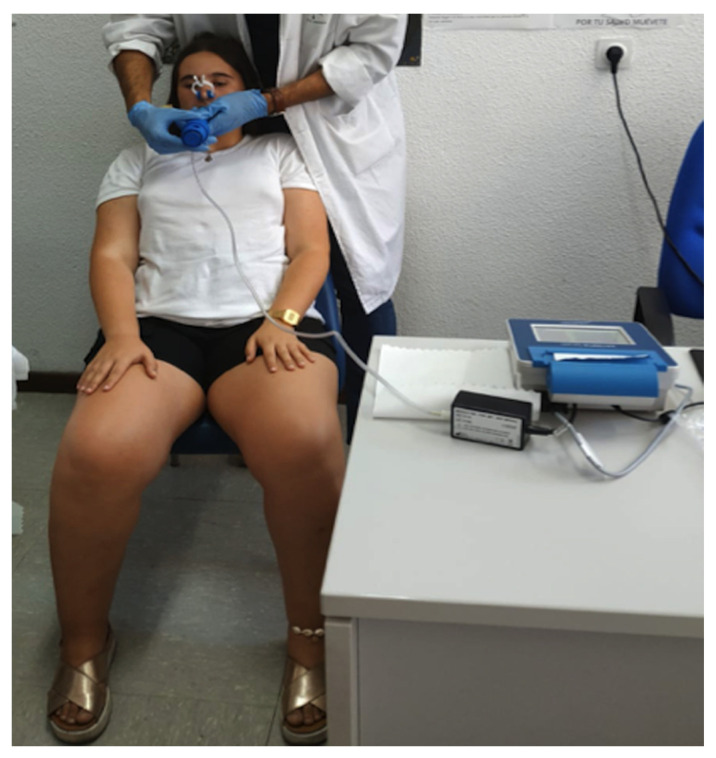
Trained physiotherapist and AIS patient using a peak inspiratory mouth pressure monitor.

Inspiratory muscle strength measurements were performed indirectly on the basis of maximum inspiratory pressure (MIP). A peak inspiratory mouth pressure monitor was used. With respect to lung function (respiratory volumes), forced vital capacity (FVC) and forced expiratory volume in the first second (FEV_1_) were measured using a digital inspirometer (Datospir Thouch, Sibelmed, Barcelona, Spain). These measurements were recorded by a previously trained physiotherapist (Figure 3). Three measurements of each variable were taken, and the best result was recorded, in line with the protocol established by the American Thoracic Society [20].

### 2.4. Data Analysis

Data were described by means and standard deviation for continuous variables and by frequencies and percentages for categorical variables. The Kolmogorov–Smirnov test was used to evaluate the normality of the data, and the Levene test was used for homoscedasticity. To measure the relationships between continuous variables, the Pearson-R correlation coefficient was used as all of them presented normal distribution. Differences in the respiratory variables between the treatment groups were determined by means of an analysis of the covariance, where the treatment group was the factor, the respiratory parameters were the result variables, and the height and degrees of curve measured on the basis of that Cobb angle were the covariates. The coefficient of determination R2 was used as an effect size measure. According to Cohen [21], R2 can be classified as insignificant when it is less than 0.02, small if it is between 0.02 and 0.15, medium if it is between 0.15 and 0.35, and large if it is greater than 0.35. Data analysis was conducted using the statistical package for social sciences version 21 (SPSS Inc., Chicago, IL, USA) and the statistical program MedCalc^®^ Statistical Software version 19.6 (MedCalc Software Ltd., Ostend, Belgium, https://www.medcalc.org; 2020). The confidence level was set at 95% (*p* < 0.05).

## 3. Results

The sociodemographic, clinical, and anthropometric characteristics of the participants are shown in Table 1. Fifteen participants were recruited and evaluated, eleven patients were female and four were male. Eight patients underwent conservative treatment (four exercises and four orthotic), and seven received no intervention.

The correlation analysis (Table 2) showed a statistically significant relationship between height, measured in centimeters, with the reference MIP (cmH_2_O) and FVC (liters) (*r* = 0.650 and *r* = 0.673; *p* < 0.01). The weight variable, measured in kg, also correlated significantly with the reference MIP (cmH_2_O) (*r* = 0.727; *p* < 0.01). The degrees of the curve, measured using the Cobb technique, significantly correlated with the variable FVC % (*r* = −0.648; *p* < 0.01).

**Figure 4:**
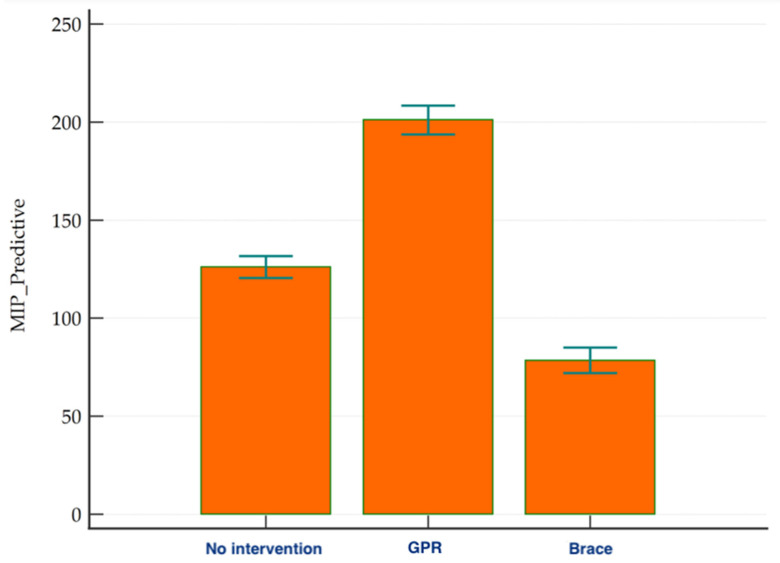
Strength scores for the different groups (GPR: Global Postural Reeducation; MIP: maximum inspiratory pressure measured in cmH_2_O).

With respect to the different types of conservative management, the strength scores were highest for the GPR treatment, followed by no intervention, with the lowest scores being obtained for the orthotic brace (Figure 4). Statistically significant differences were observed, corrected for the height and magnitude of the curve, in the predictive MIP (% cmH_2_O) (*p* < 0.05). The results can be seen in Table 3.

## 4. Discussion

In this study, it was observed that adolescents under GPR treatment presented greater inspiratory muscle strength when compared to those subjected to a brace or to no intervention. However, we did not find differences between the different cases with respect to lung function and the lack of measurements before the treatments must be considered.

In general, analyzing the entire sample, our patients presented higher values for inspiratory muscle strength (98.4 cmH_2_O and 133.4% predictive), when compared to previously published data for this population (data not presented in tables). Yagci et al. [22] studied 27 adolescents with AIS and found a predictive MIP of 48%, which is well below normal. Saraiva et al. [23] observed values between 49 and 61 cmH_2_O. The differences observed between such studies may be due, on the one hand, to the measurement procedure, or on the other to the ethnic characteristics of the population. Regarding the procedure, the values achieved for MIP will be different if the measurement is made on the basis of the functional residual capacity or on the basis of the residual volume. 

The analysis by type of treatment subgroup revealed that those patients who underwent GPR presented higher values than those in the no intervention group or the orthotic group. These differences were maintained even when the size of the curve and the height of the patients were adjusted. Although there is little scientific evidence regarding the benefits of the GPR method on respiratory variables, in clinical practice, the focus is on the respiratory system [24]. Studies performed in healthy subjects or in other patient populations have shown positive effects on respiratory muscle strength and thoracic mobility in healthy individuals [15,16]. Moreno et al. [15] demonstrated an improvement in inspiratory and expiratory muscle strength and thoracoabdominal mobility of sedentary young males. They observed increases of 40.0 cmH_2_O in MIP and 50.0 cmH_2_O in MEP in the experimental group after 16 sessions of GPR. In addition, they found an increase in thoracoabdominal expansibility in terms of the circumference values obtained at the axillary, xiphoid and abdominal level, measured using the cirtometry technique [15].

The GPR method states that respiratory alterations are the result of excessive shortening of the respiratory musculature [24]. Several factors are associated with this shortening, including stress, respiratory disease, muscle weakness, and inappropriate posture. All of the postural exercises involved in the GPR method permit respiratory muscle stretching. Muscle shortening results from modifications in the contraction proteins and metabolism of the mitochondria, with a reduction in the number of sarcomeres and an increase in the deposition of connective tissue so that the soft tissue loses elasticity [25]. Sarcomere shortening during activation is achieved by relative gliding of actin filaments over myosin filaments [25]. Stretching a muscle fiber causes a serial increase in the number of sarcomeres and a better interaction between actin and myosin filaments, by virtue of the increased functional length of the muscle. As a consequence, an increase in muscle strength associated with stretching and a loss of muscle strength associated with muscle shortening [26,27].

With respect to lung function, we did not find significant differences between the groups. The GPR method improves chest expansibility [15], and a significant association between chest expansion and vital capacity has been demonstrated [20]. However, research about the effect of GPR on lung function is scarce, especially in spinal diseases. Studies in adolescents with scoliosis treated with GPR could not be located, nonetheless some interesting ones on ankylosing spondylitis were considered. Although Durmus et al. [17] and Coksevim et al. [28] achieved better results treating ankylosing spondylitis with GPR than using a conventional exercise program regarding to FVC, FEV_1_ and PEF parameters, González-Medina et al. recently conducted a meta-analysis on GPR in ankylosing spondylitis showing that there is no evidence which suggests that GPR is better than other exercise considering respiratory parameters [29]. Though Lomas-Vega et al. determined that GPR is useful in spinal disorders [30], more research is needed to clarify its effects in respiratory function of these patients.

Some limitations of this study must be considered when interpreting the results. Firstly, we used a small sample, and the generalizability of the results to other children with AIS is limited. Secondly, the retrospective case series nature of our design does not allow causal relationships to be established. For this reason, future studies should explore prospective designs to assess the causal relationship with a larger and more diverse population. In this line, a randomized controlled trial following CONSORT guideline will be held in the future. In addition, we recommend the inclusion of functional measures related to respiratory variables, such as the degree of dyspnea or tolerance to effort in activities of daily living.

## 5. Conclusions

The results of this case series show a possible relation in which those patients who underwent treatment with the GPR method present greater inspiratory muscle strength (regardless of the size of the curve and height), when compared with the no intervention and brace cases. Lung function did not exhibit any differences between the different cases. Nevertheless, further studies are needed to confirm the generalizability of the results.

## Figures and Tables

**Table 1:** Sociodemographic characteristics.

N	Sex	Age (Year)	Height (cm)	Weight (Kg)	Body Mass Index (Kg/m^2^)	Main Curve Degrees	Type	Treatment
1	F	12	1.48	42.0	19.17	17	King IV	Brace
2	M	14	1.71	52.0	17.78	16	King IV	No intervention
3	M	13	1.74	57.0	18.83	13	King IV	No intervention
4	F	14	1.51	81.0	35.52	12	King IV	Brace
5	F	14	1.65	62.0	22.77	16	King I	GPR
6	F	10	1.45	40.0	19.02	10	King II	GPR
7	F	14	1.67	44.0	15.78	27	King I	Brace
8	F	13	1.61	48.0	18.52	21	King II	GPR
9	F	12	1.44	42.0	20.25	18	King I	No intervention
10	F	13	1.54	44.0	18.55	10	King II	No intervention
11	M	14	1.67	45.5	16.31	10	King IV	No intervention
12	M	13	1.69	55.0	19.26	10	King II	No intervention
13	F	14	1.68	73.0	25.86	12	King I	No intervention
14	F	16	1.54	48.5	20.45	37	King II	Brace
15	F	16	1.65	64.0	23.50	16	King II	GPR
Total	F = 73.3% M = 26.7%	13 ± 12 (m ± SD)	1.60 ± 0.10 (m ± SD)	53.2 ± 12.2 (m ± SD)	20.77 ± 4.86 (m ± SD)	16 ± 7 (m ± SD)	I = 26.7% III = 40.0% IV = 33.3%	No intervention = 46.7% GPR = 26.7% Brace =26.7%

Abbreviations: F, female; M, male; m, median; SD, standard deviation; GPR, Global Posture Reeducation.

**Table 2:** Pearson-R correlation between respiratory parameters and morphological and sociodemographic variables.

	Age (Year)		Height (cm)		Weight (Kg)		Body Mass Index (Kg/m^2^)		Main Curve Degrees	
	Coef	*p*	Coef	*p*	Coef	*p*	Coef	*p*	Coef	*p*
MIP	−0.131	0.643	0.113	0.689	0.229	0.412	0.318	0.248	−0.266	0.338
MIP ref	0.290	0.294	0.650	0.009 **	0.727	0.002 **	0.215	0.442	−0.293	0.289
MIP (% pred)	−0.112	0.691	0.029	0.919	0.141	0.615	0.286	0.302	−0.098	0.729
FVC (L)	0.224	0.422	0.673	0.006 **	0.485	0.067	0.007	0.980	−0.400	0.140
FVC %	−0.228	0.414	0.418	0.121	−0.028	0.922	−0.193	0.490	−0.648	0.009 **
FEV_1_ (L)	0.207	0.459	0.426	0.113	0.465	0.081	0.229	0.413	−0.185	0.510
FEV_1_ %	−0.033	0.908	0.227	0.417	0.158	0.573	0.172	0.541	−0.251	0.367
FEV_1_/FVC	0.345	0.208	−0.004	0.990	0.347	0.205	0.393	0.147	0.338	0.217
FEV_1_/FVC %	0.252	0.364	0.065	0.819	0.342	0.212	0.365	0.181	0.249	0.370
PEF (L/S)	0.259	0.351	0.304	0.270	0.454	0.089	0.286	0.302	−0.045	0.873
PEF %	0.207	0.460	0.135	0.633	0.412	0.127	0.404	0.135	−0.053	0.850

Abbreviations: MIP, maximum inspiratory pressure measured in cmH_2_O; ref, reference; %pred, percentage predicted; FVC, forced vital capacity; FEV_1_, forced expiratory volume 1 s; PEF, peak expiratory flow; L/S, liters/second; Coef, coefficient; *p*, *p*-value. ** *p* < 0.01.

**Table 3:** Mean difference in treatment groups.

	No Intervention (*n* = 7)		GPR (*n* = 4)		Brace (*n* = 4)		Ancova	Effect Size
	Mean	SD	Mean	SD	Mean	SD	*p*-Value	Eta-2
MIP	97.00	47.06	142.75	49.90	56.50	20.98	0.103	0.366
MIP ref	77.43	9.62	70.00	6.68	73.50	9.61	0.108	0.359
MIP (% pred)	126.11	57.30	201.12	55.22	78.46	33.10	0.045 *	0.463
FVC (L)	3.57	0.78	2.17	1.22	2.77	0.37	0.146	0.319
FVC %	94.14	7.78	65.25	36.68	79.00	3.92	0.147	0.318
FEV_1_ (L)	2.82	0.85	1.94	1.21	1.98	0.87	0.510	0.126
FEV_1_ %	88.14	20.46	66.25	36.99	65.00	26.23	0.336	0.196
FEV_1_/FVC	79.05	14.97	89.16	12.18	69.57	27.24	0.216	0.264
FEV_1_/FVC %	93.29	17.78	102.75	13.57	80.75	30.72	0.239	0.249
PEF L/S	4.26	1.95	3.63	2.85	3.01	1.72	0.741	0.058
PEF %	66.43	29.06	61.00	42.43	50.00	31.18	0.589	0.101

Abbreviations: MIP, maximum inspiratory pressure; Ref, reference; %pred, percentage predicted; FVC, forced vital capacity; FEV_1_, forced expiratory volume 1 s; PEF, peak expiratory flow; L/S, liters/second; SD, standard deviation; GPR, Global Posture Reeducation. * *p* < 0.05.

## Data Availability

The data presented in this study are available on request from the corresponding author.
